# Primary growth hormone insensitivity and psychomotor delay

**DOI:** 10.1002/ccr3.1349

**Published:** 2018-01-18

**Authors:** Inma Castilla‐Cortazar, Julieta Rodríguez De Ita, Gabriel A. Aguirre, Joel Rodríguez‐Rivera, Mariano García‐Magariño, Irene Martín‐Estal, Óscar Flores‐Caloca, Carlos Diaz‐Olachea

**Affiliations:** ^1^ Escuela de Medicina Tecnologico de Monterrey Monterrey Mexico; ^2^ Fundacion de Investigacion HM Hospitales Madrid Spain

**Keywords:** GH insensitivity, GH resistance, GH/IGF‐1 axis, IGF‐1, Laron Syndrome

## Abstract

We report a case of short stature irresponsive to growth hormone (GH) replacement therapy. Low GH response to provocative tests and undetectable IGF‐1 levels had suggested GH deficiency, while response to therapy indicated GH insensitivity. Molecular evaluation of the GH/IGF‐1 axis should be performed in these cases to improve diagnosis and therapy.

## Introduction

Several decades ago, Zvi Laron presented the case of 22 children with growth retardation 1 and normal growth hormone (GH) levels, condition thereafter named as Laron Syndrome and later termed primary GH insensitivity (GHI).

With technological advances, it could be clarified that GH itself was genetically and structurally normal 1. Moreover, exogenous recombinant human (rh) GH administration did not show any response or provoked a rise in serum insulin‐like growth factor 1 (IGF‐1) 1, which is secreted by the liver following GH stimulation. It was not until 1984 that “GH resistance” per se was proven, when Laron, using I^125^rGH, appreciated no attachment to liver membranes in contrast to healthy controls 1. This was further confirmed when Laron cloned the GH receptor (GHR) from two of his patients, revealing a partial gene deletion 2. Since the rise of the polymerase chain reaction technique, numerous mutations in the GHR gene have been identified 1.

The characteristic features of Laron syndrome patients are dwarfism (reaching sizes of −4 to −8 standard deviation score 1), obesity with dyslipidemia (due to metabolic derangements reaching in some cases fatty liver 1), small genitalia in boys, increased lower/upper body ratio (denoting short limbs) in females, and severe hypoglycemia in children that can later develop glucose intolerance and further type 2 diabetes and cardiovascular disease 1. Head configuration presented small face with a protruding forehead, and saddle nose together with a lowered head circumference 1. These patients also showed high pitched voice and sparse hair. At the time of the original report, Zvi Laron diagnosed these children as dwarfs with insulin‐induced hypoglycemia 1. The typical phenotype usually varies with the type of mutation associated and the degree of GH resistance; thus, the clinical features of these patients are not always the classic clinical manifestation of Laron Syndrome described by Zvi Laron, further hindering diagnosis.

Epidemiological data are hard to stablish as the majority of cases remain undiagnosed due to treatment costs or failure to identify, that is, diagnosed with idiopathic short stature. Nonetheless, to date the mayor cohorts of Laron Syndrome patients have been reported in Israel with approximately 70 patients, Ecuador with over 250 patients (the largest cohort and nearly all bearing the E180 splice mutation 3), UK (multiple ethnic origins 4), Chile, Brazil, and one US patient of Mexican origin 1, 5.

Treatment with rhIGF‐1 for Laron Syndrome, children in doses ranging from 150 to 220 μg/kg/day (improved tolerance was reported when administered with a meal rather than twice daily 1) resulted in rapid height, brain, head circumference, and organ growth 1, 6, 7. However, controversy persists regarding metabolic outcomes of such treatment, where it has shown to benefit metabolism by slightly reducing adiposity and lipidemia, improving glucose and lipid profiling, and insulin sensitivity 1, 8. Still, patients regain adiposity and lipidemia even reaching marked obesity at the end of puberty 1. It is important mention that, in Mexico, GHI is a singular pathology that lingers undiagnosed in ordinary practice. Almost all such patients continue their development without no treatment, thus developing metabolic syndrome, diabetes, hypertension, and cardiovascular disease in their adulthood. For this reason, it is really important to diagnose and treat all patients with GHI, preferably at the beginning of childhood to avoid the aforementioned pathologies.

Since Laron's discovery, a myriad of new mutations in different proteins has been reported that they directly or indirectly impact the GH/IGF‐1 axis. At the time Laron found the syndrome bearing his name, Mendelian or monogenic inheritance was the most accepted theory. This inheritance model proposed that single mutations in isolated proteins could be (and sometimes are) causative of disease. However, in this area, such model often described syndromes (3M, Tiger, Noonan…) with minimally divergent features and one common symptom: short stature. This may be explained because all such affected proteins truncated the GH/IGF‐1 axis. Now that we have a better knowledge of those pathways and that sequencing has allowed us to better understand these routes and their genes, all of these syndromes have been placed under the term “GHI.” This is because generally the resulting mutation impacts GH postreceptor signaling 9, 10, 11.

Thereby, the concept of GHI has been greatly widen since the advent of widespread sequencing, giving insight into a more complete map of mutations or variants that, together, could give a GHI phenotype. Leaving behind Mendelian inheritance, we now know that a sum of variants, mutations and the individual's genetic/epigenetic context can give rise to such diseases in the so‐called polygenic conditions. For this reason, today we can better diagnose and identify short stature causes instead of coining idiopathic causes for it.

## Case Presentation

A 3‐year‐old Mexican male with short stature presented to our department for genetic and complementary evaluation due to rhGH‐unresponsive short stature. His family history included short stature, hyperthyroidism, arterial hypertension, osteoporosis, diabetes, hypertension, and renal insufficiency.

He was born at 38 weeks by cesarean with 3500 g (75th percentile) and 50 cm (50th percentile) from nonconsanguineous healthy parents of the same ethnic origin. His mother noticed that the patient could not hold the head by himself. He sat down with 7–8 months and started crawling with 12 months. At 3 months, he was diagnosed with cow's milk protein allergy treated with rice infant formula. Ordinary pediatric revision at 7 months of age revealed no abnormalities in renal, cranial, thyroid, and urinary profiles.

Throughout his childhood, the patient presented short stature and developmental delay (head control at 5 months, walk at 3 years old), and so he was evaluated. Results showed a normal thyroid profile, normal renal ultrasonography, and normal head computed tomography scan (CT scan). However, the audiometry confirmed a precochlear auditive dysfunction, and so he was diagnosed with a developmental delay and treated with neurorehabilitation.

When the patient reached 2 years of age, short stature persisted being diagnosed with GH deficiency by a pediatric endocrinologist, who commenced with rhGH treatment for 6 months without any response.

### Investigation

**Figure 1:**
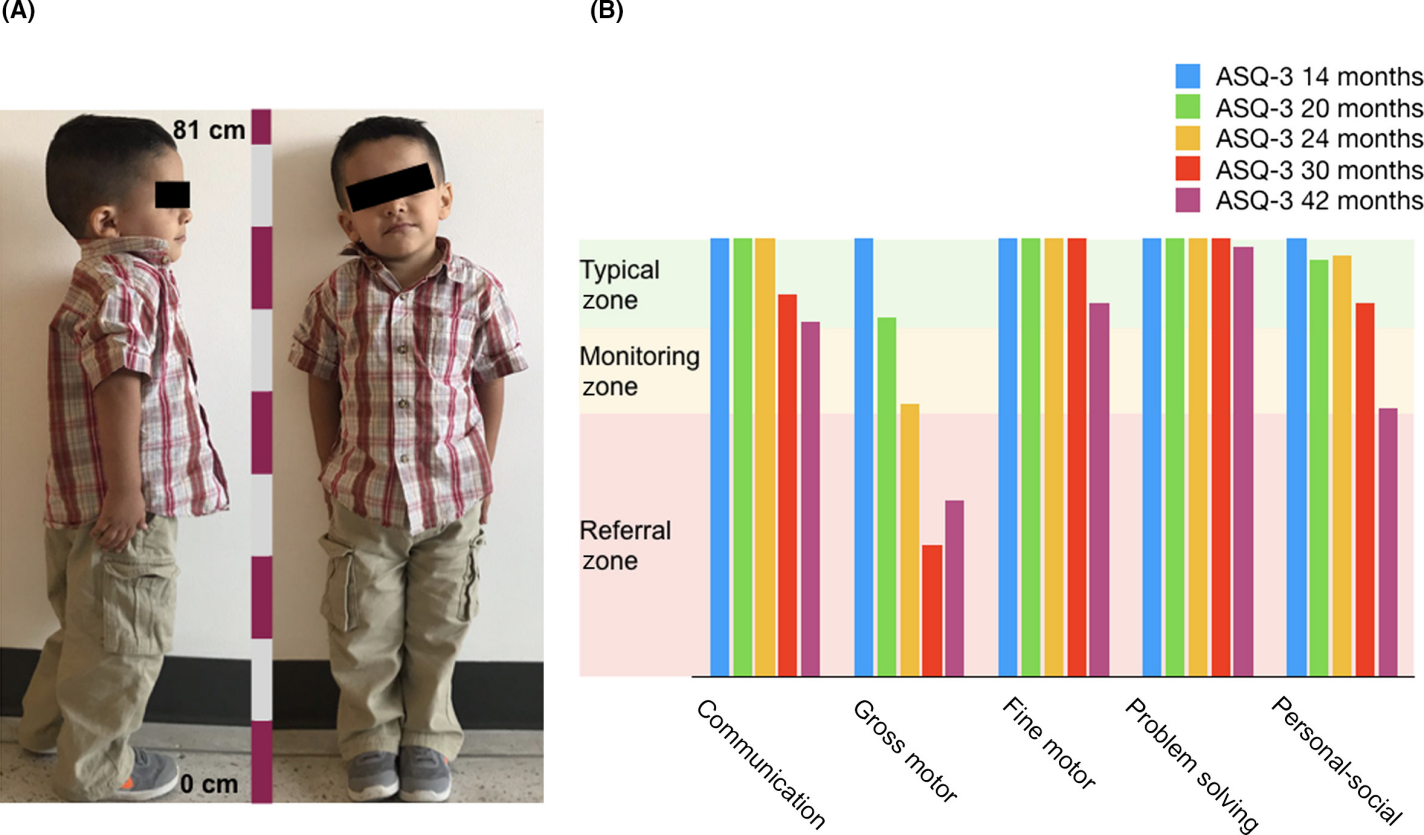
(A) Physical examination of the patient. Three‐year‐old male, stature of 81.5 cm and a weight of 11.4 kg. Evident signs of psychomotor alterations. (B) Ages & Stages Questionnaires^®^, Third Edition (ASQ‐3™) Comparison Chart. The figure represents scores for the different areas of the test (communication, gross motor, fine motor, problem solving, and personal–social) among a follow of 28 months (from month 14 to month 42) of the patient. Results show a marked impairment in the gross motor area since the 24th month of age.

Physical examination (Fig. 1A) revealed a 3‐year‐old male with an apparently correct chronological age, 81.5 cm of height (−2 standard deviations (SD) below 3th percentile for age), and weighted 11.4 kg (−2 SD below 3th percentile). He exhibited a prominent forehead (cephalic diameter of 51 cm, 50th percentile; thoracic diameter of 51 cm) as well as symmetric and eutrophic limbs (abdominal diameter of 47 cm, hip diameter 55 cm, right arm length 25, left 25 cm, right leg length 33 cm, and left 33 cm) with puffy hands. No dental, skin, or nail abnormalities were noticed. Genital examination denoted a Tanner stage I boy. An echocardiogram was performed which indicated a normal heart function. Ages and stages^®^ questionnaires denoted a gross motor and personal–social area deficiency (Fig. 1B), suggesting a psychomotor delay.

Laboratory tests displayed undetectable IGF‐1 (<25 ng/mL, age range corrected for Tanner scale 53–332 ng/mL) and low basal GH with no clonidine stimulation (basal, 0.83 ng/mL; 30 min after stimulation, 0.55 ng/mL; 60 min, nondetectable; 90 min, 0.74 ng/mL; 120 min, 0.28 ng/mL; reference: basal up to 3 ng/mL; following stimulation, an elevation over 5 ng/mL is expected).

### Molecular diagnosis

Genetic evaluation was achieved by next‐generation sequencing (Illumina MiSeq Sequencer, CA) using a Trusight One kit (Illumina, CA). Results revealed two important exonic variants within conserved regions (see Table 1). The first one is a homozygous triple (‐CAA) in‐frame deletion in MAP3K1, which codes for Mitogen Activated Kinase Kinase Kinase 1 (MAPKKK1), reported as of unknown clinical significance. The second variant is a heterozygous multiple‐nucleotide polymorphism in CUL7, which encodes Cullin‐7 (an E3 ubiquitin ligase); although considered benign by prediction algorithms, it has not been previously reported.

**Table 1:** Identified mutations by next‐generation sequencing

Gene	Variant	Chr	Coordinate	Type	Quality	GQX	Alt Var Freq	Transcript	Consequence	Amino Acids	Sift	PolyPhen	dbSNP ID	MAF
*MAP3K1*	TCAA>TCAA/T	5	56177848	Deletion	3070	132	99.07	NM_005921.1	In‐frame deletion	ST/S	–	–	rs5868032	0.245
*CUL7*	TT>TT/CC	6	43014298	mnp	198	198	41.11	NM_001168370.1	Missense variant	Q/R	Tolerated (0.86)	Benign (0)	–	0

Chr, chromosome; Alt Var Freq, alternative variant frequency, dbSNP ID, single‐nucleotide polymorphism database ID; MAF, minor allele frequency; mnp, multiple‐nucleotide polymorphism.

## Discussion

Here, we present a patient with GHI who showed delayed growth and was unresponsive to GH treatment. As there is considerable controversy around GH‐stimulation tests and the diagnosis of GH deficiency 12 and in view of the low IGF‐1 levels of the patient, we performed genetic molecular diagnosis. This revealed that there were no genetic alterations in the GH axis, while there were instead other alterations associated with GHI.

Recently, it has been characterized in an experimental model that IGF‐1 deficiency alone is associated with brain edema, oxidative damage, hypocellularity, learning alterations, and altered gene expression pattern for genes encoding proteins involved in cerebral extracellular matrix, and tight junctions 13. In this line, clinical studies have found a role for IGF‐1 in brain development, neuronal survival, neurogenesis, angiogenesis, excitatory and inhibitory neurotransmission, regulation of food intake, and cognition 14 as well as linked to several neuropathies during adulthood 15. Therefore, herein we suggest a plausible causal relationship between GHI and psychomotor neurodevelopmental delay.

It is of vital importance to treat GHI patients with rhIGF‐1 (if no IGF‐1 insensitivity is found) and not with rhGH, as they are resistant to it and more problems could arise, like abnormal extremities growth and metabolic derangements. Never‐treated IGF‐1‐deficient patients (i.e., from IGF‐1 deficiency conditions like GHI, fetal growth restriction, polycystic ovarian syndrome, and hepatic cirrhosis) may also develop metabolic problems leading to metabolic syndrome and ultimately to type 2 diabetes 8, 16, 17, 18. In this case, IGF‐1 treatment could palliate neurodevelopmental and growth delay, also protecting against metabolic derangements that usually appear around puberty in Laron Syndrome patients 16, 17, 18. We encourage to study the full GH/IGF‐1 axis including its regulatory elements to rule out a possible GHI before coining a diagnosis of idiopathic short stature or just lightly administering rhGH treatment with its concomitant complications if the patient is GH‐resistant.

Concerning molecular diagnosis, it has been shown that activating mutations in the MAPK pathway leading to nuclear factor kappa beta (NF*κβ*) overactivation inhibits the Janus kinase (JAK)/STAT pathway 11, 19, in what was classically considered “rasopathies.” STAT5b is the most important transcription factor for IGF‐1 13. Hence, such variant could be hindering GHR‐dependent IGF‐1 expression. Also, MAP3K1 has E3 ubiquitin ligase activity 20, belonging to the SCF complex involved in GHR turnover 11, which could indicate that the variant may also be altering GHR's presence. Even though such variant possesses penetration within the population, in the context of a *CUL7* mutation, it could be causing a growth restriction phenotype in a polygenic pattern condition. Typically, Cullin‐7 is associated with the so‐called 3‐M syndrome, being a E3 ubiquitin ligase at the proteasome in charge, among others, in the turnover and splicing of GHR and several growth factors receptors (including IGF1R) and, more importantly, in the degradation of IRS1, crucial for IGF‐1 receptor signaling 21. Also, our patient does not present any 3‐M syndrome clinical feature, like triangular facies, prominent heels, thoracic, abdominal, and skeletal abnormalities. Therefore, summing all of the above, alterations in MAP3K1 inhibit JAK/STAT pathway that may lead to decreased IGF‐1, IGFBP3, and ALS transcription. MAP3K1 also acts as a E3 ubiquitin ligase together with *CUL7*, which is also a ubiquitin ligase. They both regulate growth factor receptor splicing, activation, and turnover. All of such could lead to the GHI phenotype described herein. Although GH levels and provocation test fail to coincide with classical GHI, the patient's phenotype is not congruent with GH deficiency. Consistently, it is widely known that GH provocation test often is subject to false negatives and lacks reproducibility 22. With this in mind, and taking into account the patient's lack of response to GH treatment, aided by the lack of GH deficiency variants/mutations, a diagnosis of GHI was made.

## Conclusions

Clinical manifestations, biochemical results, and molecular diagnoses confirm another case of GHI without GHR mutations in a 3‐year‐old Mexican patient. We propose rhIGF‐1 substitutive treatment as soon as possible.

## Ethical Approval

All procedures were carried out under a written patient's informed consent without any coercion and approval from Clinical Ethics Committee from Tecnológico de Monterrey.

## Authorship

ICC: clinical diagnosis and case presentation, proportioned and stablished the techniques to the patient diagnosis and contributed to paper writing and review; JRDI: clinical procedures for diagnosis of the patient and the presentation of the case; MGM, GAA, JRR, and IME: contributed to sample processing and literature review; GAA: contributed by establishing the molecular diagnosis of the patient, figure designing and paper writing; OFC: introduced the patient; and lastly, CDO: performed the analytical analyses.

## Conflict of Interest

Authors declare that there is no financial or other potential conflict of interest that could be perceived as prejudicing the impartiality of the research reported.
